# Discovery of Quality Markers of Nucleobases, Nucleosides, Nucleotides and Amino Acids for Chrysanthemi Flos From Different Geographical Origins Using UPLC–MS/MS Combined With Multivariate Statistical Analysis

**DOI:** 10.3389/fchem.2021.689254

**Published:** 2021-08-05

**Authors:** Xiangwei Chang, Zhenyu Zhang, Hui Yan, Shulan Su, Dandan Wei, Sheng Guo, Erxin Shang, Xiaodong Sun, Shuangying Gui, Jinao Duan

**Affiliations:** ^1^ College of Pharmacy, Anhui University of Chinese Medicine, Hefei, China; ^2^ Institute of Pharmaceutics, Anhui Academy of Chinese Medicine, Hefei, China; ^3^ Jiangsu Collaborative Innovation Center of Chinese Medicinal Resources Industrialization, Nanjing University of Chinese Medicine, Nanjing, China; ^4^ Jiangsu Hexiang Juhai Modern Agricultural Industrialization Co., Ltd, Yancheng, China

**Keywords:** Chrysanthemi Flos, geographical origins, nutritional ingredients, quality markers, UPLC-MS/ MS, Support Vector Machines model

## Abstract

Nucleobases, nucleosides, nucleotides and amino acids, as crucial nutrient compositions, play essential roles in determining the flavor, function and quality of Chrysanthemi Flos. The quality of Chrysanthemi Flos from different geographical origins is uneven, but there have been no reports about the screening of their quality markers based on nutritional ingredients. Here, we developed a comprehensive strategy integrating ultra performance liquid chromatography coupled with triple-quadrupole linear ion-trap tandem mass spectrometry (UPLC–MS/MS) and multivariate statistical analysis to explore quality markers of Chrysanthemi Flos from different geographical origins and conduct quality evaluation and discrimination of them. Firstly, a fast, sensitive, and reliable UPLC–MS/MS method was established for simultaneous quantification 28 nucleobases, nucleosides, nucleotides and amino acids of Chrysanthemi Flos from nine different regions in China. The results demonstrated that Chrysanthemi Flos from nine different cultivation regions were rich in the above 28 nutritional contents and their contents were obvious different; however, correlation analysis showed that altitude was not the main factor for these differences, which required further investigation. Subsequently, eight crucial quality markers for nine different geographical origins of Chrysanthemi Flos, namely, 2′-deoxyadenosine, guanosine, adenosine 3′,5′-cyclic phosphate (cAMP), guanosine 3′,5′-cyclic monophosphate (cGMP), arginine, proline, glutamate and tryptophan, were screened for the first time using partial least squares discriminant analysis (PLS-DA) and the plot of variable importance for projection (VIP). Moreover, a hierarchical clustering analysis heat map was employed to intuitively clarify the distribution of eight quality markers in the nine different regions of Chrysanthemi Flos. Finally, based on the contents of selected eight quality markers, support vector machines (SVM) model was established to predict the geographical origins of Chrysanthemi Flos, which yielded excellent prediction performance with an average prediction accuracy of 100%. Taken together, the proposed strategy was suitable to discover the quality markers of Chrysanthemi Flos and could be used to discriminate its geographical origin.

## Introduction

Chrysanthemi Flos, the dried flower head of *Chrysanthemum morifolium* Ramat (Compositae), is a medicinal and edible cognate plant and has been widely used for thousands of years as a diet in China, especially flower tea for healthcare and traditional Chinese medicine (TCM) (Chen et al., 2021). As an essential TCM, Chrysanthemi Flos is commonly used for “dissipating cold”, “removing heat and toxins from the body”, and “brightening eyes”. Modern pharmacological studies have reported broad pharmacological effects for Chrysanthemi Flos, such as anti-inflammatory (Ding et al., 2016), antimicrobial (Kuang et al., 2018), antioxidative (Cui et al., 2012), antiadipogenic (Lee et al., 2014), antihypertensive (Lv et al., 2013), antidiabetic (Nishina et al., 2019), anticancer (Xie et al., 2009), cardiovascular protective (He et al., 2012) and neuroprotective effects (Yang et al., 2017), etc. Moreover, significant amounts of constituents have been found in Chrysanthemi Flos, including flavonoids, phenolic acids (Lin and Harnly, 2010), volatile oils (Luo et al., 2017), polysaccharides (Zheng et al., 2015), triterpenoids (Ragasa et al., 2005), inorganic elements (Park and Kwon, 1997), nucleosides and amino acids (Chang et al., 2020), the former three types of which are regarded as the major components and likely responsible for these aforementioned activities (Yuan et al., 2020).

According to different cultivation regions, Chrysanthemi Flos have been divided into Boju (BJ), Chuju (CJ), Gongju (GJ), Huaibaiju (HBJ), Sheyanghangbaiju (SYHBJ), Tongxianghangbaiju (TXHBJ), Fubaiju (FBJ), Qiju (QJ), and Jiaju (JJ) in the marketplace, exhibit specific geographical indication, and they are indiscriminate in medicinal and tea use. Notably, BJ, CJ, GJ, HBJ, and Hangbaiju (SYHBJ and TXHBJ) among them have been officially recorded in the Chinese Pharmacopoeia (2020 edition) under the same item of “Juhua” as standard varieties of Chrysanthemi Flos. Although the evaluation criteria of Chrysanthemi Flos from different cultivation regions are the same in the Chinese Pharmacopoeia, the prices of Chrysanthemi Flos from different geographical origins vary greatly in the market due to their quality differences. Moreover, previous studies have shown that geographical environments greatly influence the chemical compositions and quality of Chrysanthemi Flos (Xie et al., 2012; Du et al., 2015; Han et al., 2015). Currently, only chlorogenic acid, 3,5-O-dicaffeoylquinic acid and luteolin-7-O-β*-*D-glucoside are quantified as marker compounds for the quality control of Chrysanthemi Flos in the Chinese Pharmacopoeia. To evaluate the quality of Chrysanthemi Flos from different geographical origins, many holistic chemical profiling methods have been carried out, such as liquid chromatography-mass spectrometry (LC-MS) for the analysis of flavonoids and phenolic acids (Ding et al., 2016; Nie et al., 2018; Zhang et al., 2018), and gas chromatography-mass spectrometry (GC-MS) for the analysis of volatile compounds (Luo et al., 2017; Nie et al., 2018; Zhang et al., 2018). Obviously, flavonoids, phenolic acids, and volatile oils have been the main focus for most studies. However, as far as we know, there have been no reports on the quality evaluation of Chrysanthemi Flos from different geographical origins based on the nutritional compounds such as nucleobases, nucleosides, nucleotides, and amino acids.

Nucleobases, nucleosides, nucleotides, and amino acids are crucial nutritional and functional compounds, and they exhibit diverse bioactivities including immuno-regulatory, antioxidative, anti-obesity, antihypertensive, and anticancer effects (Wu, 2013; Chang et al., 2020). Besides health benefit function, previous studies have demonstrated that some of these nutrient compositions were positively correlated with the tea flavor and quality of tea (Yang et al., 2018; Yu and Yang, 2019). For example, Anji white tea is a popular high-quality tea in China because of its abundant amino acids (Yu and Yang, 2019). More importantly, nucleobases, nucleosides, nucleotides and amino acids have been screened as quality markers of several Chinese herbal medicines (CHMs) and functional foods, such as Mactra veneriformis (Liu et al., 2012), *Ziziphus jujuba* (Guo et al., 2013), royal jelly (Wu et al., 2015) and *Angelica sinensis* (Qu et al., 2019). In our previous study, glutamate, asparagine and aspartate were selected as key quality markers for Chrysanthemi Flos from three different flowering stages (Chang et al., 2020). Therefore, the discovery of these nutritional compounds as quality markers will facilitate the effective use and quality control of Chrysanthemi Flos from different geographical origins.

In the present study, an integrated strategy of ultra performance liquid chromatography coupled with triple-quadrupole linear ion-trap tandem mass spectrometry (UPLC–MS/MS) and multivariate statistical analysis was developed to reveal the quality markers of Chrysanthemi Flos from different geographical areas based on nutritional compounds. To start, the UPLC–MS/MS method to simultaneously quantitate 28 nucleobases, nucleosides, nucleotides, and amino acids of Chrysanthemi Flos from nine different geographical origins was established and validated in this study. On the basis of these 28 nutrient compositions, multivariate statistical methods, including principal component analysis (PCA) and partial least squares discriminant analysis (PLS-DA), were applied to discover quality markers of Chrysanthemi Flos from nine different cultivation regions. A hierarchical clustering analysis heat map was then employed to visualize the distribution of the selected quality markers in nine different geographical origins of Chrysanthemi Flos. Moreover, support vector machines (SVM) model was established to discriminate and predict the geographical origins of Chrysanthemi Flos using selected quality markers. In summary, this study provides a new strategy for quality assessment of Chrysanthemi Flos in nine different geographical origins as well as other medicinal herbs or foods using nutritional ingredients as quality markers.

## Materials and Methods

### Chemicals, Reagents, and Plant Materials

Ultrapure water (H_2_O) was purified by a Milli-Q water purification system (Millipore, Bedford, MA, United States). LC-MS grade ammonium formate and ammonium acetate were obtained from Fisher Scientific (Wyman Street, Waltham, MA, United States). LC-MS grade methanol, acetonitrile and formic acid were purchased from Merck (Darmstadt, Germany). Chemical standards of uracil (**1**), 2′-deoxyinosine (**7**), 2′-deoxycytidine (**8**) and 2′-deoxyguanosine (**9**) were purchased from Aladdin Reagent Co. Ltd. (Shanghai, China). Reference compounds of guanine (**2**), leucine (**14**), phenylalanine (**15**), tryptophan (**16**), isoleucine (**17**), methionine (**18**), valine (**19**), threonine (**20**), lysine (**21**), proline (**22**), tyrosine (**23**), glutamate (**24**) and arginine (**27**) were obtained from Huixing Biochemical Reagent Ltd. (Shanghai, China). Chemical standards of 2′-deoxythymidine (**3**), 2′-deoxyadenosine (**4**), uridine (**5**), adenosine (**6**), cytidine (**10**), guanosine (**11**), adenosine 3′,5′-cyclic phosphate (cAMP, **12**), guanosine 3′,5′-cyclic monophosphate (cGMP, **13**), glutamine (**25**), asparagine (**26**) and gamma-aminobutyric acid (GABA, **28**) were purchased from Sigma-Aldrich (St. Louis, MO, United States). The purities of all of the reference standards were >98%, determined by HPLC analysis. The chemical structures of the above compounds (**1**–**28**) are shown in Supplementary Figure 1.

**FIGURE 1:**
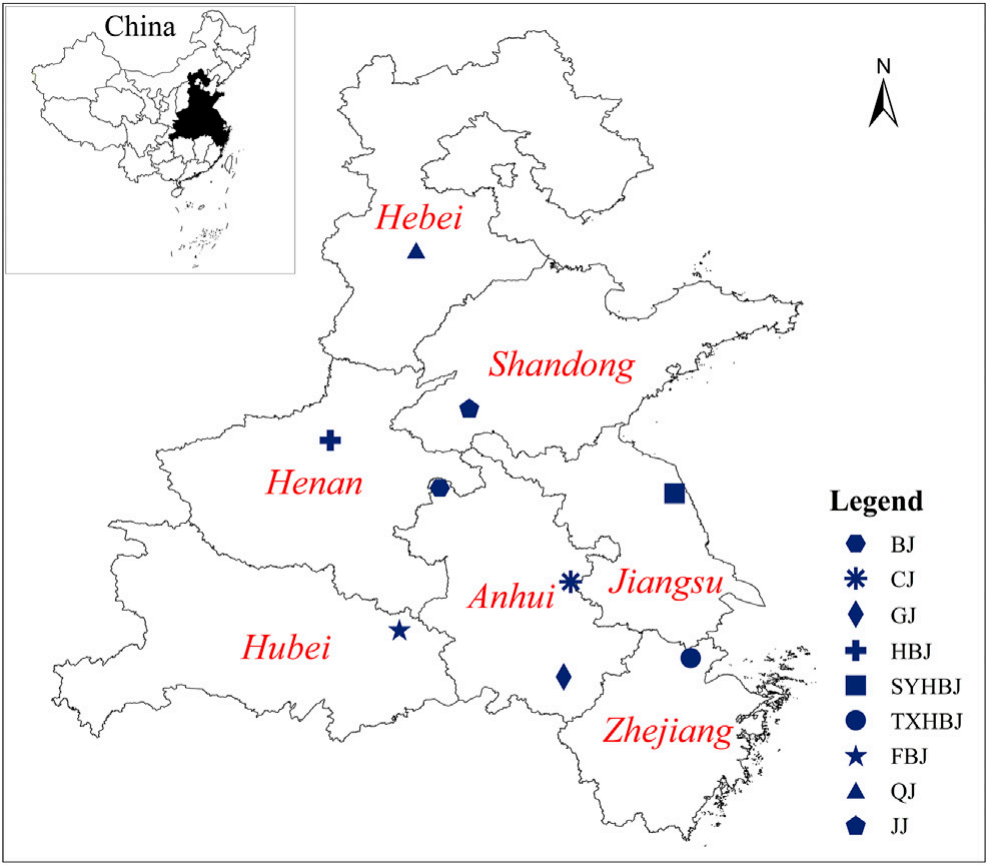
The geographical location of Chrysanthemi Flos samples collected from China.

The samples of Chrysanthemi Flos were collected from nine different geographical origins in China from October to November 2018, and the detailed information of all samples is reported in Figure 1, Supplementary Figure 2; Supplementary Table 1. Their botanical origins were identified by Professor Jin-ao Duan (Nanjing University of Chinese Medicine, Jiangsu Province, China). Voucher specimens were deposited at the Herbarium of Jiangsu Collaborative Innovation Center of Chinese Medicinal Resources Industrialization.

### Preparation of Standard Solutions

The standard stock solutions were prepared by dissolving approximately 1 mg of each reference compounds in 10 ml of H_2_O. According to the results of preliminary experiments, an appropriate amount of the above 28 standard stock solutions was mixed and then diluted with water to obtain reference compound mixture working solutions with eight different non-zero concentration levels for constructing the calibration curves. Three independent replicated analyses at each concentration level were executed in this study using UPLC–MS/MS. Prior to analysis, all the standard solutions were filtered through a 0.22 µm cellulose membrane filter and stored at 4°C.

### Preparation of Sample Solutions

The dried samples were pulverized to homogeneous powders (80 mesh). Each dried sample powder (1.0 g) precisely weighed was added to a 50 ml glass-stoppered conical flask containing 40 ml of distilled water. All of the mixtures were accurately weighed and sonicated (100 Hz, 25°C) for 60 min, and then water was added to compensate for the water lost during extraction. After centrifugation (13,000 r/ min, 15 min), the supernatants were kept at 4°C and filtered through 0.22 µm cellulose membrane filters before injection into the UPLC–MS/MS system for analysis. 10 replicates of Chrysanthemi Flos at each cultivation region were prepared to improve the reliability.

### UPLC–MS/MS Analysis Conditions

Chromatographic analysis was performed on a Waters ACQUITY UPLC™ system (Waters, Milford, MA, United States), which was equipped with a binary solvent manager, a column manager and an automatic sampler. The ACQUITY BEH amide column (100 mm × 2.1 mm, 1.7 µm) was applied with the column temperatures set at 30°C. The temperature of the automatic sampler was maintained at 10 °C. The binary mobile phase was composed of A (0.2% formic acid, 5 mM ammonium formate and 5 mM ammonium acetate in H_2_O) and B (0.2% formic acid, 1 mM ammonium formate and 1 mM ammonium acetate in acetonitrile). UPLC linear gradient elution conditions were: 0–3 min, 10% A; 3–9 min, 10–18% A; 9–15 min, 18–20% A; 15–16 min, 20–46% A; 16–18 min, 46% A. The injection volume was 1 μL, and the flow rate was set at 0.4 ml/ min.

Mass spectrometry was carried out by a QTRAP 6500^+^ triple quadrupole linear ion-trap mass spectrometer equipped with a TurboV™ ion source (Applied Biosystems SCIEX, CA, United States) operating in positive ion mode. The parameters of QTRAP-MS/MS analysis were set as follows: ion-spray voltage 5500 V, source temperature 400°C, curtain gas 40 psi, nebulizer gas (GS1, nitrogen) 40 psi, and auxiliary heater gas (GS2, nitrogen) 40 psi. The collisionally activated dissociation (CAD) was set to a medium level. Based on the selected parent and daughter ions, multiple reaction monitoring (MRM) mode was applied to analyze 28 analytes (1–28). The raw data of all Chrysanthemi Flos samples were acquired by the Analyst 1.6.3 software (Applied Biosystems SCIEX, CA, United States).

## Method Validation

The validation of UPLC–MS/MS method established in this study was performed by evaluating linearity, limits of detection (LOD), limits of quantification (LOQ), precision, repeatability, stability, recovery, and matrix effects according to our previous study (Chang et al., 2020).

### Calibration Curves, LOD and LOQ

The mixed standard solution was diluted to a series of solutions with at least six appropriate concentrations in duplicate to make calibration curves. Then linear regression was constructed by plotting the peak areas versus the corresponding concentration of each analyte. The LOD and LOQ for each analyte under the present UPLC–MS/MS conditions were determined at signal-to-noise ratios (S/N) of 3:1 and 10:1, respectively. The peak height divided by the background noise value was calculated as the S/N.

### Precision, Repeatability and Stability

Intra- and inter-day variations were chosen to assess the precision of the method. For intra-day precision test, the mixed standard solutions were analyzed for six times within 1 day, while for inter-day precision test, the mixed standard solutions were examined by repeating the experiments during three consecutive days. To evaluate the repeatability, six sample solutions were independently prepared from the same batch of Chrysanthemi Flos samples and analyzed in parallel. To test the stability of the sample solutions, one of the Chrysanthemi Flos sample solutions mentioned above was stored at 25°C and determined in various periods (0, 4, 8, 12, 24, and 48 h), respectively. All these variations are expressed as relative standard deviation (RSD).

### Recovery

A recovery test was conducted to evaluate the accuracy of this method (Guo et al., 2013; Li et al., 2013; Zhao et al., 2018). The test was performed by spiking known quantities of the 28 standards with high, middle and low levels to a certain amount of Chrysanthemi Flos sample which had been analyzed in the repeatability test. Then, the spiked samples were extracted, processed and quantified on the basis of the methods described above, and triplicate experiments were performed on each level. The average recovery rate was calculated by the following formula: recovery (%) = [(observed amount—original amount)/spiked amount] × 100% (Guo et al., 2013; Wu et al., 2015; Zhao et al., 2018).

### Matrix Effects

The matrix effect was defined as the ion suppression or enhancement in the process of analyte ionization (Matuszewski et al., 2003; Guo et al., 2013). The slope comparison method was used to evaluate the latent interfering effect from co-eluting matrix constituents on the ESI response (Chen et al., 2012; Guo et al., 2013). The sample extracts were spiked with appropriate amounts of standards, similar to the procedure for the apparent recovery measurements based on the recovery parameters described above, and were used to construct standard addition calibration curves. Then, the slopes of the calibration curves of the standard addition experiments were compared with the slopes obtained from the pure aqueous standards at the same concentration levels. Matrix effects are expressed as a matrix factor by the equation: matrix factor (MF) = slope matrix/slope solvent, MF = 1 indicates no significantly matrix effects, MF < 1 denotes ion suppression, MF > 1 indicates ion enhancement (Chen et al., 2012; Silvestro et al., 2013). Before injection, the sample extracts were stored at 4°C for 24 h to allow interaction between the analytes and the matrix of the sample.

### Multivariate Statistical Analysis

In this study, 90 Chrysanthemi Flos samples and the contents of 28 target compounds in these samples constructed a data matrix with 90 rows and 28 columns. The 90 × 28 data matrix was made into a table and subjected to PCA and PLS-DA using SIMCA-P software (Ver.13, Umetrics, Umea, Sweden). The plot of variable importance for projection (VIP) was applied to screen and find quality markers of nutritional compounds for Chrysanthemi Flos from nine different geographical origins. A one-way analysis of variance (ANOVA) was conducted using SPSS 20.0 software (IBM SPSS Statistics, Chicago, IL, United States). Pearson correlation analysis, hierarchical clustering analysis heat map, F-test, partial F-test and Cramer-von Mises (CVM) test were performed in the R routine (Desharnais et al., 2017a; Wei and Simko, 2017). Furthermore, (SVM) model for the classification and prediction of geographical origins of Chrysanthemi Flos samples was established under Matlab R2017a software environment (Mathworks, Natick, United States).

## Results and Discussion

### Development of UPLC–MS/MS Analysis Conditions

**FIGURE 2:**
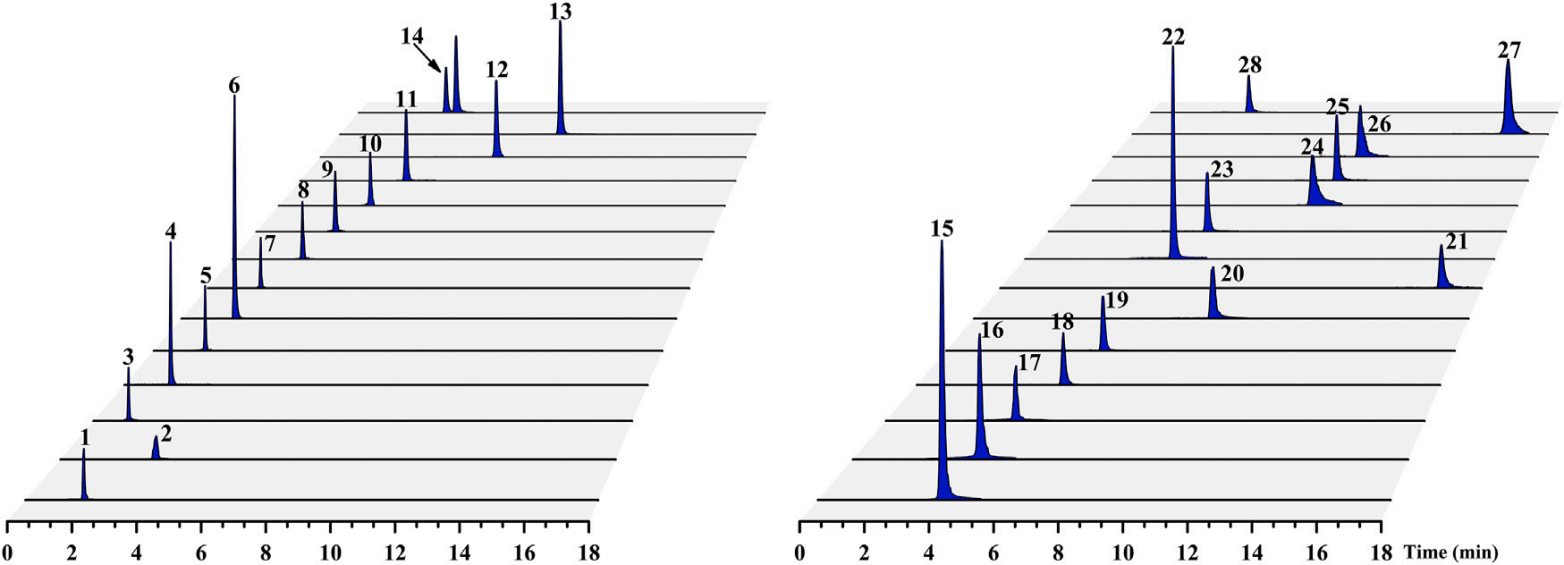
Typical UPLC–MS/MS chromatograms of the 28 target compounds for the Chrysanthemi Flos sample (SYHBJ-S43). The numbering of the 28 target compounds in the chromatograms is the same as in Table 1.

The nucleobases, nucleosides, nucleotides, and amino acids are the compounds with high polarity, which are easily separated in high ratio of aqueous mobile phase and have poor retention property on reversed-phase column. Our previous study showed that the peak capacity, retention ability and resolution of the ACQUITY BEH amide column (100 mm × 2.1 mm, 1.7 µm) were better than those of the ACQUITY BEH C18 (100 mm × 2.1 mm, 1.7 µm) and ACQUITY HSS T3 (100 mm × 2.1 mm, 1.8 µm) columns for these hydrophilic compounds of Chrysanthemi Flos under the same chromatographic condition (Chang et al., 2020). Therefore, the ACQUITY BEH amide column was chosen for this study. Additionally, different mobile phase modifiers, including different concentrations of ammonium formate, ammonium acetate, formic acid and their combination, were investigated to increase the separation and improve the peak shapes of the 28 target analytes in our preliminary experiment (Chang et al., 2020). Consequently, a mixture consisting of A (aqueous solution containing 5 mM ammonium formate, 5 mM ammonium acetate and 0.2% formic acid) and B (acetonitrile solution containing 1 mM ammonium formate, 1 mM ammonium acetate and 0.2% formic acid) was considered as the most suitable mobile phase system. The typical chromatograms of the Chrysanthemi Flos sample (SYHBJ-S43) and reference compound mixture solution are presented in Figure 2 and Supplementary Figure 3, respectively.

As for MS/MS detection conditions, both positive and negative ion modes were first tested. It was found that positive ion mode had lower background noise, higher sensitivity and intensity than negative ion mode for the 28 target analytes, which made it easier to detect the lower content of analytes in the Chrysanthemi Flos extracts. Therefore, positive ion mode was adopted for the 28 target compounds detection and characterization. Due to the mutual interference may happen between the compounds producing ions with similar *m/z*, the MRM mode was utilized in this study to improve the selectivity and sensitivity for these target compounds (Yao et al., 2015). Then, the declustering potential (DP), collision energy (CE), cell exit potential (CXP) and entrance potential (EP) parameters were optimized for each analyte to obtain the most selective and specific precursor/product ion pairs by injecting individual standard solutions in direct infusion mode. The MRM parameters of 28 investigated compounds are listed in Table 1.

**TABLE 1:** MRM parameters of 28 target compounds detected on the UPLC–MS/MS.

NO.	Analytes	RT (min)	Precursor ion (*m/z*)	Product ion (*m/z*)	DP (V)	EP (V)	CE (V)	CXP (V)
1	Uracil	1.82	112.959	70.1	101	10	21	8
2	Guanine	3.09	152.040	109.9	111	10	27	12
3	2′-deoxythymidine	1.17	243.052	126.9	11	10	13	14
4	2′-deoxyadenosine	1.57	252.021	136.0	26	10	19	14
5	Uridine	1.81	245.119	113.0	21	10	13	12
6	Adenosine	1.91	268.023	136.1	31	10	23	14
7	2′-deoxyinosine	1.98	253.024	136.9	11	10	11	16
8	2′-deoxycytidine	2.70	228.034	112.1	1	10	11	12
9	2′-deoxyguanosine	3.09	268.066	152.0	11	10	13	16
10	Cytidine	3.66	244.161	112.0	31	10	15	12
11	Guanosine	4.35	284.056	152.0	1	10	17	16
12	cAMP	7.46	329.971	136.0	121	10	31	16
13	cGMP	9.55	345.954	152.0	96	10	25	18
14	Leucine	3.87	132.069	86.2	11	10	13	10
15	Phenylalanine	3.88	166.097	120.1	1	10	17	14
16	Tryptophan	4.11	205.089	146.1	26	10	25	16
17	Isoleucine	4.32	132.069	69.0	11	10	25	8
18	Methionine	5.04	150.117	104.0	36	10	15	12
19	Valine	5.55	118.125	72.0	1	10	13	8
20	Threonine	8.69	120.128	74.0	16	10	13	8
21	Lysine	16.51	147.110	84.0	61	10	23	10
22	Proline	5.64	116.167	70.0	21	10	19	10
23	Tyrosine	6.24	182.097	136.1	41	10	19	16
24	Glutamate	9.76	148.051	84.1	66	10	21	10
25	Glutamine	10.07	147.072	84.1	51	10	23	10
26	Asparagine	10.48	133.111	87.1	1	10	13	10
27	Arginine	16.29	175.110	70.1	86	10	27	8
28	GABA	4.34	104.022	87.1	1	10	17	52

### UPLC–MS/MS Method Validation

The proposed UPLC–MS/MS method for the quantitation of 28 target analytes was validated to determine the linearity, LOD, LOQ, intra-day and inter-day precisions, repeatability, stability, recovery, and matrix effect. As shown in Supplementary Table 2, the *F*-test yielded *p*-values < 0.05 for the 28 target compounds, indicating that all data-point distributions were heteroscedastic. Then, a weighting factor of 1/x^2^ was used in the calibration process (Gu et al., 2014; Alladio et al., 2020). The *p*-values obtained from the partial *F*-test were >0.05 for the 28 target compounds, and a linear calibration model was, therefore, adopted for them. Moreover, the correlation coefficient values (*r* ^2^) were better than 0.9972 for all 28 target compounds, indicating good linear regressions between the investigated compounds’ concentrations and their peak areas within the test ranges. CVM test produced *p*-values > 0.05 for all 28 target analytes, suggesting that all calibration curves were considered validated (Desharnais et al., 2017a; Desharnais et al., 2017b). The overall LODs and LOQs were in the range of 0.03–22.62 ng/ ml and 0.10–86.65 ng/ ml, respectively, which revealed that the established analytical method was sensitive enough for quantitative determination of these target analytes. Moreover, the relative standard deviation (RSD) values of intra- and inter-day variations of the 28 target analytes were less than 3.62 and 6.24%, respectively (Supplementary Table 3). The RSD values of the repeatability and stability were all not more than 6.84%, and all analytes of Chrysanthemi Flos sample solutions remained stable for 48 h. Additionally, the overall recoveries were in the range from 94.30 to 104.75% with RSD values less than 5.60% for all analytes. The slope ratio values of the matrix curves to the neat solution curve were between 0.91 and 1.06, implying that there were no significant ion suppression or enhancement between the 28 target compounds under current conditions. These results demonstrated that the developed UPLC–MS/MS method was sensitive, repeatable, and accurate for the determination of the 28 nucleobases, nucleosides, nucleotides and amino acids in nine different cultivation regions of Chrysanthemi Flos.

### Distribution of 28 Target Analytes in Chrysanthemi Flos From Nine Different Geographical Origins

The UPLC–MS/MS method developed in this study was used for the simultaneous quantification of 2 nucleobases (1–2), 9 nucleosides (3–11), 2 nucleotides (12–13) and 15 amino acids (14–28) in the Chrysanthemi Flos samples collected from nine different geographical origins. Among the 15 amino acids measured, 8 are essential amino acids (14–21), 6 are non-essential amino acids (22–27), and 1is non-protein amino acid (28). Table 2 shows the contents of each target compound (1–28) and the total concentrations of the analyzed compounds in nine different cultivation regions of Chrysanthemi Flos. The results demonstrated that almost all of the Chrysanthemi Flos samples from different geographical areas were rich in 28 nutrient compositions including nucleobases, nucleosides, nucleotides and amino acids, but they had obvious differences in the contents. The total contents of the 28 target compounds in Chrysanthemi Flos samples from nine regions varied from 3.19 mg/ g in TXHBJ to 27.43 mg/ g in CJ, and they were ranked in the order of CJ > QJ > GJ > SYHBJ > BJ > JJ > HBJ > FBJ > TXHBJ. In addition, the total contents of 15 amino acids in Chrysanthemi Flos from nine different geographical origins were much higher than that of 13 nucleobases, nucleosides and nucleotides, and the former was about 2.10–17.41 times that of the latter. The total contents of 13 nucleobases, nucleosides and nucleotides were relatively higher in FBJ with the content of 1.58 mg/ g compared to the other geographical origins of Chrysanthemi Flos. In contrast, the total contents of 15 amino acids were relatively higher in CJ with the content of 25.94 mg/ g compared with the other geographical origins of Chrysanthemi Flos. Notably, the total contents of six non-essential amino acids in nine different geographical origins of Chrysanthemi Flos were significantly higher than that of eight essential amino acids, and the former was approximately 3.58–7.21 times that of the latter. To sum up, from nutritional and functional points of view, Chrysanthemi Flos from different geographical origins might be promising natural sources for the manufacture of different functional products such as tea and other beverages according to the characteristics of their nutritional ingredients.

**TABLE 2:** Contents (μg/g) of 28 target compounds in Chrysanthemi Flos from nine different geographical origins.

Analytes^a^	BJ	CJ	GJ	HBJ	SYHBJ
1	99.59 ± 6.35 ^b^	133.82 ± 3.74	134.16 ± 9.31	78.86 ± 5.55	48.44 ± 4.31
2	24.05 ± 0.71	62.08 ± 0.82	41.23 ± 0.95	21.06 ± 0.68	2.02 ± 0.10
3	31.81 ± 1.46	144.00 ± 3.15	105.44 ± 4.68	48.44 ± 5.70	6.63 ± 0.39
4	22.28 ± 1.09	3.68 ± 0.42	0.29 ± 0.05	0.19 ± 0.03	6.26 ± 0.16
5	290.40 ± 5.75	430.91 ± 23.20	382.11 ± 13.82	216.11 ± 8.55	54.99 ± 2.13
6	244.48 ± 6.82	0.64 ± 0.06	2.06 ± 0.16	nd ^c^	332.54 ± 10.66
7	1.49 ± 0.87	0.70 ± 0.07	0.37 ± 0.02	0.46 ± 0.05	0.54 ± 0.04
8	14.74 ± 0.88	65.98 ± 1.05	38.80 ± 1.79	14.03 ± 1.53	2.12 ± 0.13
9	30.27 ± 1.44	138.32 ± 1.80	77.65 ± 4.57	30.99 ± 3.41	5.92 ± 0.25
10	212.23 ± 5.02	425.91 ± 15.21	315.64 ± 10.29	176.67 ± 6.69	8.14 ± 0.28
11	255.83 ± 9.57	74.75 ± 21.35	28.00 ± 5.38	17.31 ± 3.93	57.26 ± 2.38
12	3.27 ± 0.07	3.88 ± 0.10	3.56 ± 0.10	3.25 ± 0.09	252.04 ± 7.59
13	4.51 ± 0.24	4.65 ± 0.25	4.65 ± 0.29	3.97 ± 0.12	331.86 ± 8.56
14	265.65 ± 4.99	271.36 ± 6.69	232.43 ± 10.81	107.69 ± 3.01	107.08 ± 3.20
15	384.10 ± 6.64	572.86 ± 14.80	508.25 ± 12.81	276.39 ± 7.53	256.67 ± 7.45
16	187.71 ± 4.34	384.04 ± 9.25	225.67 ± 5.12	121.45 ± 2.84	149.67 ± 5.36
17	361.04 ± 8.28	476.15 ± 14.35	370.59 ± 12.42	182.74 ± 7.01	220.12 ± 9.83
18	39.24 ± 1.42	38.76 ± 1.06	38.20 ± 1.95	23.53 ± 1.71	13.76 ± 1.05
19	397.17 ± 8.64	571.50 ± 15.96	427.86 ± 26.20	217.08 ± 6.80	256.01 ± 6.88
20	420.96 ± 11.14	738.12 ± 28.18	581.75 ± 20.38	270.63 ± 5.22	330.17 ± 12.28
21	206.04 ± 11.11	423.32 ± 20.59	205.46 ± 7.95	91.60 ± 7.29	38.03 ± 4.00
22	1866.11 ± 69.84	7750.39 ± 188.55	5151.42 ± 259.89	1918.32 ± 120.34	5330.43 ± 143.24
23	159.54 ± 3.42	312.07 ± 7.41	291.57 ± 4.37	133.35 ± 7.42	99.33 ± 3.24
24	310.41 ± 8.64	849.91 ± 23.31	576.10 ± 19.92	275.16 ± 19.76	1193.30 ± 31.99
25	2998.38 ± 100.90	6202.38 ± 219.70	1592.31 ± 66.89	802.51 ± 27.47	1063.66 ± 29.19
26	1869.63 ± 87.17	4519.08 ± 148.51	2462.52 ± 99.86	1294.18 ± 56.78	987.40 ± 24.38
27	885.42 ± 30.02	2346.51 ± 54.00	1534.37 ± 64.26	539.37 ± 53.62	1012.74 ± 23.52
28	404.32 ± 7.38	482.79 ± 19.93	489.00 ± 32.82	212.70 ± 13.02	111.97 ± 2.90
TNS ^d^	1103.53 ± 22.52	1284.89 ± 47.80	950.37 ± 31.82	504.20 ± 26.96	474.41 ± 13.76
TNN	1234.95 ± 26.18	1489.32 ± 47.84	1133.98 ± 28.14	611.32 ± 25.59	1108.77 ± 29.38
TEA	2261.92 ± 46.08	3476.11 ± 100.04	2590.22 ± 82.92	1291.11 ± 31.47	1371.51 ± 38.45
TNEA	8089.48 ± 269.52	21980.34 ± 587.85	11608.28 ± 413.39	4962.89 ± 249.28	9686.85 ± 211.73
TAA	10755.72 ± 316.70	25939.24 ± 697.00	14687.49 ± 523.28	6466.70 ± 279.70	11170.33 ± 246.89
TNA	11990.67 ± 339.66	27428.56 ± 737.72	15821.48 ± 520.34	7078.02 ± 301.46	12279.10 ± 273.06

Analytes ^a^	TXHBJ	FBJ	QJ	JJ
1	33.31 ± 4.72 ^b^	81.76 ± 6.35	160.50 ± 4.23	21.37 ± 1.85
2	0.93 ± 0.05	2.91 ± 0.19	41.35 ± 0.33	0.26 ± 0.04
3	8.48 ± 0.47	16.36 ± 0.76	99.30 ± 4.63	0.54 ± 0.12
4	6.12 ± 0.38	16.53 ± 0.40	11.28 ± 0.68	2.40 ± 0.21
5	60.71 ± 5.93	213.66 ± 8.03	433.26 ± 10.13	33.60 ± 1.89
6	391.60 ± 21.89	524.43 ± 15.20	0.27 ± 0.01	187.62 ± 9.21
7	0.38 ± 0.04	0.30 ± 0.05	1.18 ± 0.19	0.25 ± 0.02
8	2.99 ± 0.23	8.78 ± 0.27	42.55 ± 1.57	0.22 ± 0.04
9	7.98 ± 0.55	19.09 ± 0.62	82.11 ± 3.59	1.71 ± 0.11
10	17.10 ± 1.37	119.96 ± 4.55	326.26 ± 2.60	14.68 ± 0.71
11	178.39 ± 16.85	472.20 ± 16.25	290.84 ± 9.85	99.47 ± 5.32
12	98.56 ± 4.27	36.23 ± 1.97	3.97 ± 0.09	144.14 ± 8.19
13	220.92 ± 11.58	68.67 ± 2.85	7.36 ± 0.19	230.97 ± 13.31
14	22.79 ± 1.97	86.79 ± 3.27	227.35 ± 2.08	53.74 ± 2.91
15	35.24 ± 2.48	157.41 ± 4.83	706.17 ± 6.12	115.55 ± 4.75
16	54.44 ± 2.62	66.43 ± 1.94	286.91 ± 2.84	174.37 ± 7.19
17	68.84 ± 3.19	140.83 ± 2.87	444.33 ± 10.61	127.65 ± 4.62
18	3.86 ± 0.65	2.97 ± 0.25	24.69 ± 1.42	3.30 ± 0.44
19	49.15 ± 3.74	148.69 ± 3.82	726.82 ± 8.26	96.56 ± 4.14
20	87.25 ± 6.21	178.67 ± 7.23	778.85 ± 13.97	185.75 ± 7.53
21	16.60 ± 2.10	44.11 ± 4.27	90.86 ± 4.01	13.15 ± 3.43
22	521.01 ± 34.07	1094.56 ± 27.25	6496.58 ± 58.83	430.64 ± 16.23
23	23.25 ± 1.75	60.55 ± 2.48	253.90 ± 3.35	58.14 ± 2.64
24	648.36 ± 47.85	1124.64 ± 25.99	512.45 ± 10.71	1026.54 ± 40.90
25	274.70 ± 20.29	964.38 ± 35.74	2825.60 ± 48.27	1522.22 ± 73.42
26	135.30 ± 17.70	300.37 ± 20.39	6822.88 ± 94.99	1187.15 ± 57.78
27	126.19 ± 8.49	398.52 ± 22.61	1308.74 ± 13.62	1329.90 ± 66.07
28	93.05 ± 1.25	196.97 ± 6.98	405.60 ± 15.19	96.00 ± 2.70
TNS^d^	673.76 ± 46.54	1391.32 ± 43.91	1287.06 ± 18.75	340.49 ± 16.94
TNN	1027.48 ± 57.69	1580.89 ± 45.81	1500.23 ± 18.50	737.22 ± 39.18
TEA	338.17 ± 15.05	825.89 ± 21.94	3285.98 ± 31.95	770.07 ± 30.89
TNEA	1728.82 ± 124.04	3943.01 ± 111.10	18220.15 ± 194.66	5554.59 ± 248.10
TAA	2160.04 ± 136.65	4965.87 ± 132.89	21911.73 ± 229.49	6420.66 ± 278.84
TNA	3187.52 ± 192.07	6546.76 ± 177.60	23411.97 ± 233.62	7157.89 ± 316.74

a The 28 analytes are the same as in Table 1. In these analytes, 1–2 are nucleobases; 3–11 are nucleosides; 12–13 are nucleotides; 14–28 are amino acids, 14–21 of which are essential amino acids, 22–27 are non-essential amino acids and 28 is non-protein amino acid.

b Mean ± SD (n = 10).

c Not detected.

d TAA, is the total contents of all amino acids; TEA, is the total contents of essential amino acids; TNA, is the total contents of TNN and TAA; TNEA, is the total contents of non-essential amino acids; TNN, is the total contents of nucleobases, nucleosides and nucleotides; TNS, is the total contents of nucleosides.

In terms of individual compounds, the remarkable differences were also observed in nine different geographical origins of Chrysanthemi Flos. The content of uridine was evidently higher than those of other nucleobases, nucleosides and nucleotides in BJ, CJ, GJ, HBJ, and QJ. The content of adenosine in SYHBJ, TXHBJ, and FBJ was obviously greater than those of other nucleobases, nucleosides and nucleotides. Furthermore, the concentration of cGMP in JJ was observed to be relatively higher than those of all the other nucleobases, nucleosides and nucleotides. For free amino acids, the contents of three of the non-essential amino acids (proline, glutamine and asparagine) were greater than 0.1% in most geographical origins of Chrysanthemi Flos, making them notably more abundant than any other amino acids. More interestingly, the content of glutamate, known as an important umami amino acid, was fairly higher (greater than 0.1%) in SYHBJ, FBJ, and JJ compared to the other geographical origins of Chrysanthemi Flos. The above results indicated that the differences in the distribution of these nutrient compositions might be the one of the reasons for the differences in reputed flavor between Chrysanthemi Flos from nine different regions.

### Pearson Correlation Analysis of the 28 Target Analytes and Elevation in Different Geographical Origins of Chrysanthemi Flos

**FIGURE 3:**
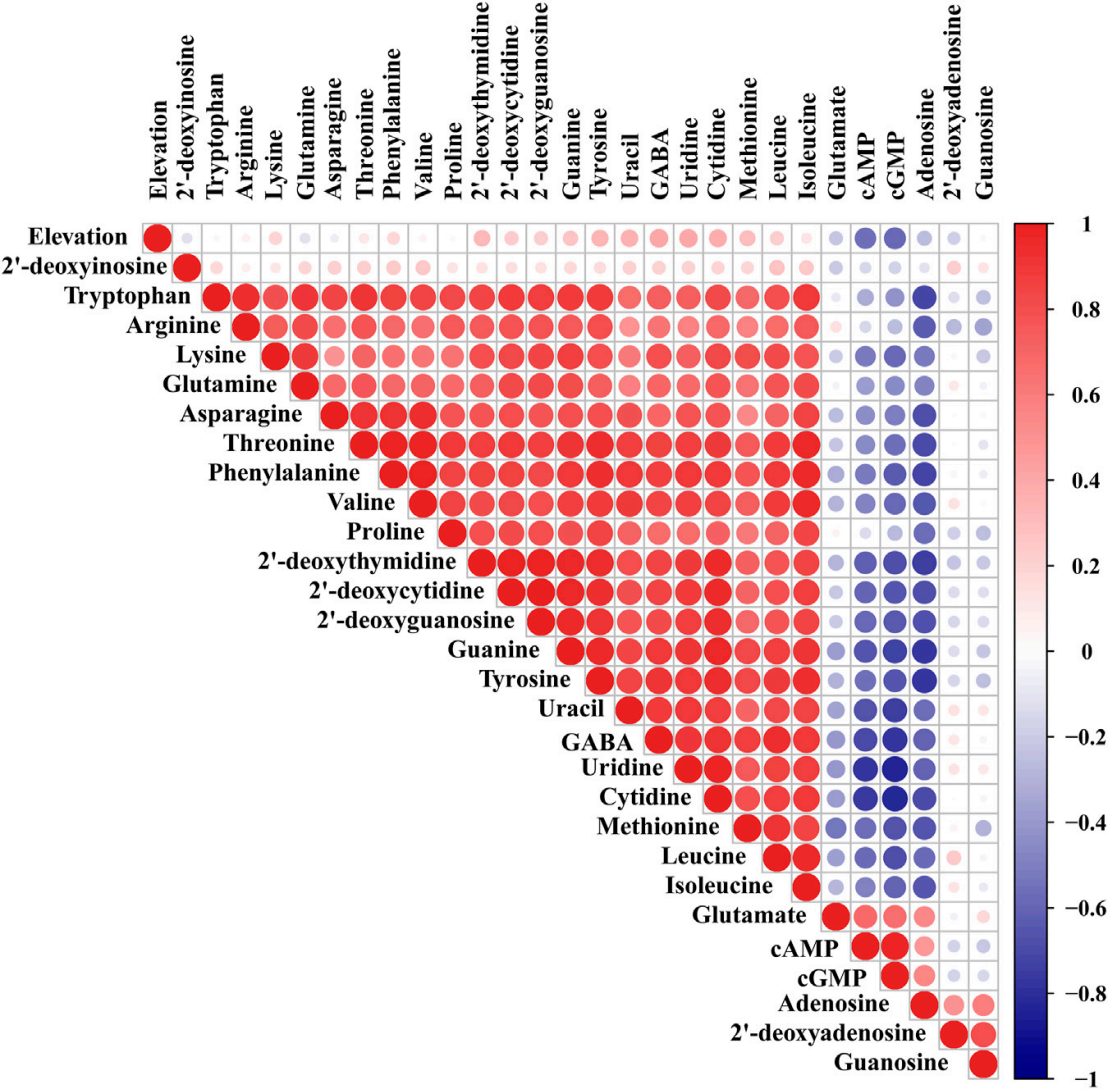
Pearson correlation analysis of the 28 target analytes and elevation in nine different geographical origins of Chrysanthemi Flos.

Pearson correlation analysis was performed on the 28 target compounds and elevation for Chrysanthemi Flos from nine different geographical origins by the R package “corrplot” (Wei and Simko, 2017). As illustrated in Figure 3, the warm and cold colours represent positive and negative correlations, respectively, and the deep colours and big circles represent large correlation coefficients and strong correlations. Strong positive correlations were observed among 2 nucleobases (uracil and guanine), 5 nucleosides (2′-deoxythymidine, uridine, 2′-deoxycytidine, 2′-deoxyguanosine and cytidine), 8 essential amino acids, 5 non-essential amino acids (proline, tyrosine, glutamine, asparagine, and arginine) and 1 non-protein amino acid (GABA), and almost all Pearson correlation coefficients were greater than 0.6. The above results indicated that the accumulation rules of these nutrients (especially amino acids) were similar. Furthermore, adenosine, cAMP and cGMP were negatively correlated with most of the target compounds investigated in this study, and the absolute values of almost all Pearson correlation coefficients were greater than 0.6. It was worthy noted that the altitude of Chrysanthemi Flos from different geographical origins varied greatly. Among them, SYHBJ was located at the lowest altitude with an elevation of 2 m, and GJ was located at the highest altitude with an elevation of 154 m. Correlation analysis results demonstrated that the elevation was negatively correlated with cAMP and cGMP, and positively correlated with uracil, uridine, cytidine, tyrosine, and GABA, but the Pearson correlation coefficients were all less than 0.6. Therefore, altitude might not be the main factor leading to the differences in nutritional components of Chrysanthemi Flos from different geographical origins, which required further investigation.

### Discovery of Quality Markers for Nine Different Geographical Origins of Chrysanthemi Flos

Multivariate statistical analysis, including PCA, and PLS-DA, was carried out based on the contents of the 28 target analytes to clarify the intrinsic similarities and differences of Chrysanthemi Flos from nine different geographical origins and further screen the potential quality markers between them. PCA, an unsupervised model, was first used to explore the underlying trends in the Chrysanthemi Flos samples from different cultivation regions after Pareto scaling with mean-centering. According to default cross-validation configurations (7-fold cross-validation), ten principal components (PCs) were significant for the PCA model. The first two principal components dominated the PCA model and accounted for 78.0% of the variation, indicating good fitness of the established PCA model. As seen in Supplementary Figure 4, ninety Chrysanthemi Flos samples were well segregated into nine groups corresponding to their cultivation regions. The results indicated that nucleobases, nucleosides, nucleotides and amino acids could be used to distinguish Chrysanthemi Flos from nine different geographical origins and had the potential to control the quality of Chrysanthemi Flos.

**FIGURE 4:**
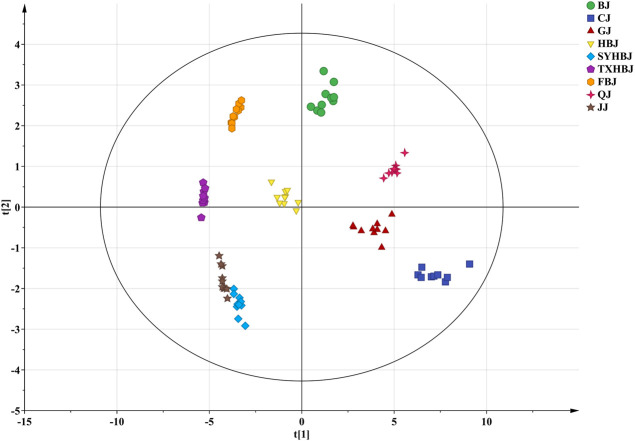
PLS-DA score plot of Chrysanthemi Flos from nine different geographical origins.

**FIGURE 5:**
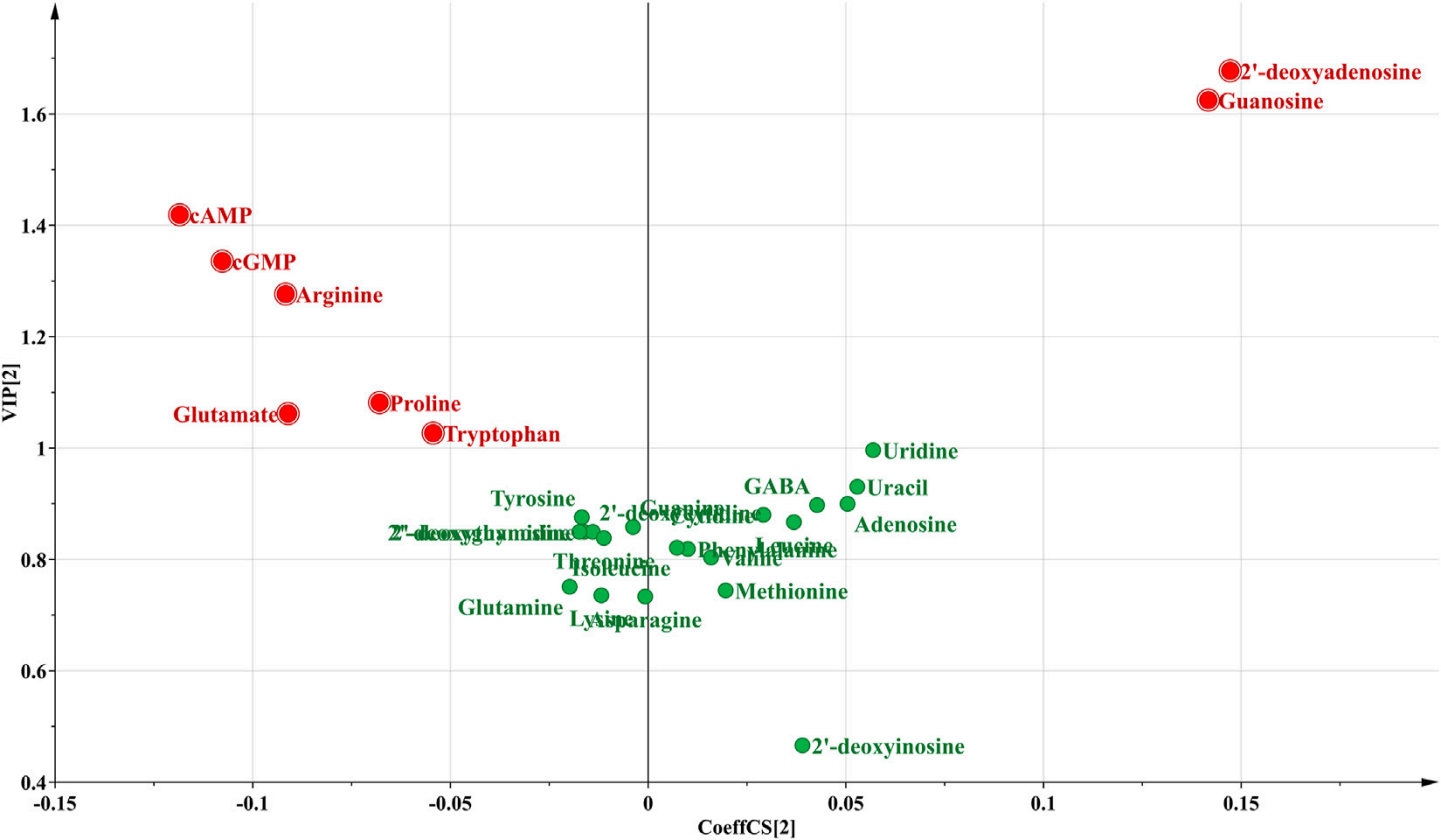
The VIP plot of 28 target compounds in nine different geographical origins of Chrysanthemi Flos.

**FIGURE 6:**
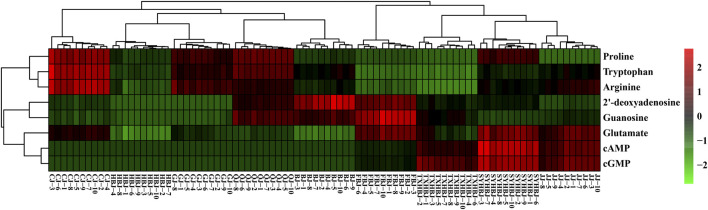
Heatmap visualization of the eight quality markers in nine different geographical origins of Chrysanthemi Flos.

Then, a supervised PLS-DA model was built to further discover the potential quality markers for nine different geographical origins of Chrysanthemi Flos using the same data as the above mentioned PCA. As shown in the two-dimensional PLS-DA score plot (Figure 4), the Chrysanthemi Flos samples in nine different geographical areas were clearly classified into nine clusters, scattering in different quadrants of the 95% Hotelling’s T^2^ ellipse, which was similar with the PCA results. Normally, the performance of a PLS-DA model was evaluated by simultaneously considering the explained variation R^2^Y (goodness of fit) and the predicted variation Q^2^ (goodness of prediction) (Chang et al., 2017; Chang et al., 2021). Nine principal components were significant for the PLS-DA and the model fit parameters were 0.928 for R^2^Y and 0.915 for Q^2^ according to 7-fold cross-validation, revealing excellent classification and predictive abilities of the PLS-DA model. Moreover, the permutation test was a method to evaluate the statistical significance of the PLS-DA model, which was subsequently used to further verify the established PLS-DA model. In our 200 rounds of random permutation test (Supplementary Figure 5), the R^2^ and Q^2^ intercepts were 0.042 and −0.381, respectively, indicating that the PLS-DA model was statistically valid and was not overfitted. Next, the potential quality markers for nine different geographical origins of Chrysanthemi Flos were selected according to the VIP plot from the PLS-DA model. Based on the VIP plot, shown in Figure 5, eight target analytes with a VIP value of >1.0, including 2′-deoxyadenosine, guanosine, cAMP, cGMP, arginine, proline, glutamate, and tryptophan according to the order of their VIP values from large to small, mainly contributed to the differentiation of Chrysanthemi Flos from nine different geographical origins and were considered as quality markers. All these eight quality markers exhibited statistically significant differences among the nine different geographical origins of Chrysanthemi Flos based on one-way ANOVA. Moreover, a hierarchical clustering analysis heat map (Figure 6) was used to intuitively display the distribution of eight quality markers in the nine different geographical origins of Chrysanthemi Flos.

To test the capability of the selected eight quality markers for the discrimination of geographical origins of Chrysanthemi Flos, a PLS-DA analysis was performed again on Chrysanthemi Flos samples from nine different cultivation regions based on these eight quality markers. The model parameters were 0.782 for R^2^Y, 0.765 for Q^2^, 0.008 for R^2^ intercept, and −0.251 for Q^2^ intercept, which means that the PLS-DA model has good fitness and prediction without overfitting (Supplementary Figure 6). As observed in Supplementary Figure 6A, ninety Chrysanthemi Flos samples were significantly and accurately classified into nine groups in terms of their geographical origins. Notably, the SYHBJ and JJ samples were also clearly separated and no longer overlapped. The results demonstrated that the screened eight nutritional compounds were robust quality markers that can clearly discriminate Chrysanthemi Flos from different cultivation regions.

### SVM for the Classification and Prediction of Chrysanthemi Flos in Different Geographical Origins

**FIGURE 7:**
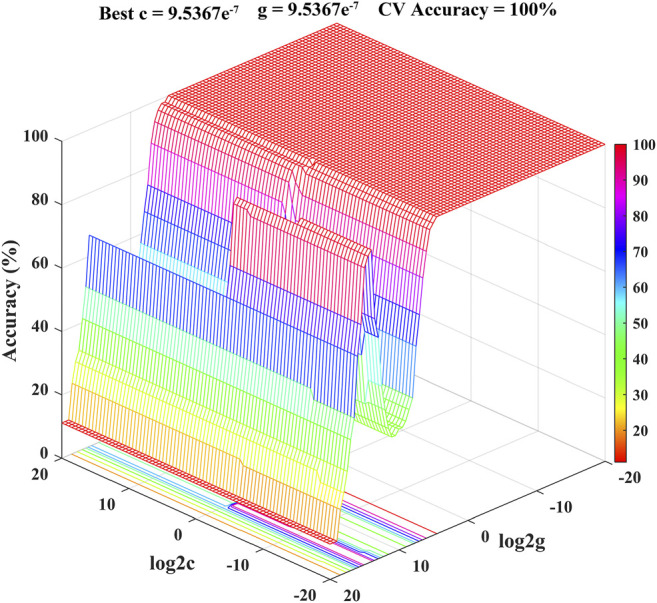
The results of the optimal combination for parameters *C* and *g* (gamma) of the SVM model.

SVM is a supervised learning model with excellent learning performance and is especially suitable for analyzing small sample data sets (Ma et al., 2016; Li et al., 2017). At present, as an effective tool, SVM has been successfully applied to the quality control of foods and Chinese herbal medicines with satisfactory classification and prediction accuracy (Wang et al., 2017; Richter et al., 2019; Jin et al., 2021). Therefore, SVM model for classification and prediction of geographical origins of Chrysanthemi Flos in this study was established using the dataset containing the contents of eight quality markers as the input vectors and the nine different regions as the output vectors. The radial basis function was selected as the kernel function to build the SVM model (Li and Wang, 2018; Li et al., 2018). At the same time, 10-fold cross-validation was performed to improve prediction accuracy and avoid over-fitting of the established SVM model (Gao et al., 2012; Wang et al., 2017). All ninety Chrysanthemi Flos samples from nine different regions were randomly classified into the calibration set (63 samples for developing the discrimination model) and prediction set (27 samples for testing the prediction accuracy of the established SVM model) using the Kennard-stone algorithm. The best combination for penalty parameter C and kernel function parameter *g* (gamma) of the SVM model was calculated using grid search method combined with 10-fold cross-validation (Li and Wang, 2018). As shown in Figure 7, the optimal parameter combination was obtained: Best *c* = 9.5367e^−7^, Best *g* = 9.5367e^−7^ and CV accuracy = 100%, indicating that the established SVM model was not over-fitting (Li et al., 2017; Li et al., 2020). As illustrated in Table 3, all Chrysanthemi Flos samples were correctly divided into nine groups corresponding to their geographical origins and the prediction accuracy reached 100%. Thus, the results strongly indicated that the developed SVM model based on eight quality markers was a powerful tool for the classification and prediction of geographical origins of Chrysanthemi Flos, further demonstrating the reliability of the screened quality markers.

**TABLE 3:** The classified and predicted results of Chrysanthemi Flos from nine different geographical origins using SVM model.

Calibration set (recognition accuracy = 100%)						Prediction set (prediction accuracy = 100%)		
Sample	Actual	Recognized	Sample	Actual	Recognized	Sample	Actual	Predicted
S2	1^a^	1	S47	5	5	S1	1	1
S4	1	1	S48	5	5	S3	1	1
S5	1	1	S50	5	5	S6	1	1
S7	1	1	S51	6	6	S12	2	2
S8	1	1	S52	6	6	S14	2	2
S9	1	1	S53	6	6	S15	2	2
S10	1	1	S54	6	6	S26	3	3
S11	2	2	S56	6	6	S27	3	3
S13	2	2	S57	6	6	S29	3	3
S16	2	2	S58	6	6	S33	4	4
S17	2	2	S61	7	7	S36	4	4
S18	2	2	S62	7	7	S40	4	4
S19	2	2	S64	7	7	S42	5	5
S20	2	2	S65	7	7	S45	5	5
S21	3	3	S67	7	7	S49	5	5
S22	3	3	S68	7	7	S55	6	6
S23	3	3	S70	7	7	S59	6	6
S24	3	3	S72	8	8	S60	6	6
S25	3	3	S73	8	8	S63	7	7
S28	3	3	S76	8	8	S66	7	7
S30	3	3	S77	8	8	S69	7	7
S31	4	4	S78	8	8	S71	8	8
S32	4	4	S79	8	8	S74	8	8
S34	4	4	S80	8	8	S75	8	8
S35	4	4	S82	9	9	S81	9	9
S37	4	4	S83	9	9	S86	9	9
S38	4	4	S84	9	9	S87	9	9
S39	4	4	S85	9	9			
S41	5	5	S88	9	9			
S43	5	5	S89	9	9			
S44	5	5	S90	9	9			
S46	5	5						

a Different number represents the different geographical origins: 1**–**Bozhou, Anhui; 2**–**Chuzhou, Anhui; 3**–**Huangshan, Anhui; 4**–**Zhengzhou, Henan; 5**–**Sheyang, Jiangsu; 6**–**Tongxiang, Zhejiang; 7**–**Macheng, Hubei; 8**–**Anguo, Hebei; 9**–**Jiaxiang, Shandong.

## Conclusion

From the perspective of nutritional ingredients, this study proposed a research strategy for screening quality markers of Chrysanthemi Flos from nine different geographical origins using UPLC–MS/MS combined with multivariate statistical analysis. A UPLC–MS/MS method capable of quantifying 28 nucleobases, nucleosides, nucleotides, and amino acids was first established, and then applied it to study the variation trend of these nutrient compositions in nine different regions of Chrysanthemi Flos. The results revealed that Chrysanthemi Flos from nine different cultivation regions were rich in nucleobases, nucleosides, nucleotides and amino acids and their contents were significantly different, but altitude was not the main reason for these differences. Next, eight quality markers for nine different geographical origins of Chrysanthemi Flos were discovered for the first time based on the above 28 nutritional compounds using PLS-DA and VIP plot. Furthermore, a heat map visualization was performed to clarify the distribution of eight quality markers. More importantly, the established SVM model based on eight quality markers showed excellent classification and prediction performance for Chrysanthemi Flos in different geographical origins. The proposed approach was helpful in elaborating more specific quality evaluation standards for Chrysanthemi Flos and provided a simple and reliable method for discovery of quality markers for other TCMs and foods.

## Data Availability

The original contributions presented in the study are included in the article/Supplementary Material, further inquiries can be directed to the corresponding authors.
