# Supplementary material for: Discovery of Quality Markers of Nucleobases, Nucleosides, Nucleotides and Amino Acids for Chrysanthemi Flos From Different Geographical Origins Using UPLC–MS/MS Combined With Multivariate Statistical Analysis
Source: Front Chem. 2021 Aug 5;9:689254. doi: 10.3389/fchem.2021.689254 (PMC8375154; doi:10.3389/fchem.2021.689254)
Supplement: Supplementary file 1 [file DataSheet1.docx]

***Supplementary Material***

**Discovery of** **Quality Markers of** **Nucleobases, Nucleosides, Nucleotides and Amino Acids for Chrysanthemi Flos from Different Geographical Origins Using UPLC–MS/MS** **Combined with Multivariate Statistical Analysis**

**Xiangwei Chang ^1,2^, Zhenyu Zhang ^3^, Hui Yan ^3,^*, Shulan Su ^3^, Dandan Wei ^3^, Sheng Guo ^3^, Erxin Shang ^3^, Xiaodong Sun ^4^, Shuangying Gui ^1,2^, Jinao Duan ^3,^***

*^1^ College of Pharmacy, Anhui University of Chinese Medicine, Hefei* *230012, China*

*^2^ Institute of Pharmaceutics, Anhui Academy of Chinese Medicine, Hefei 230012, China*

*^3^Jiangsu Collaborative Innovation Center of Chinese Medicinal Resources Industrialization, Nanjing University of Chinese Medicine, Nanjing 210023, China*

*^4^ Jiangsu Hexiang Juhai Modern Agricultural Industrialization Co., Ltd, Yancheng 224335, China*

* Corresponding authors.

*E-mail addresses*: dja@njucm.edu.cn (J.-a. Duan), yanhui@njucm.edu.cn (H. Yan).





**Figure S1.** The chemical structures of the 28 target compounds quantified by UPLC–MS/MS.


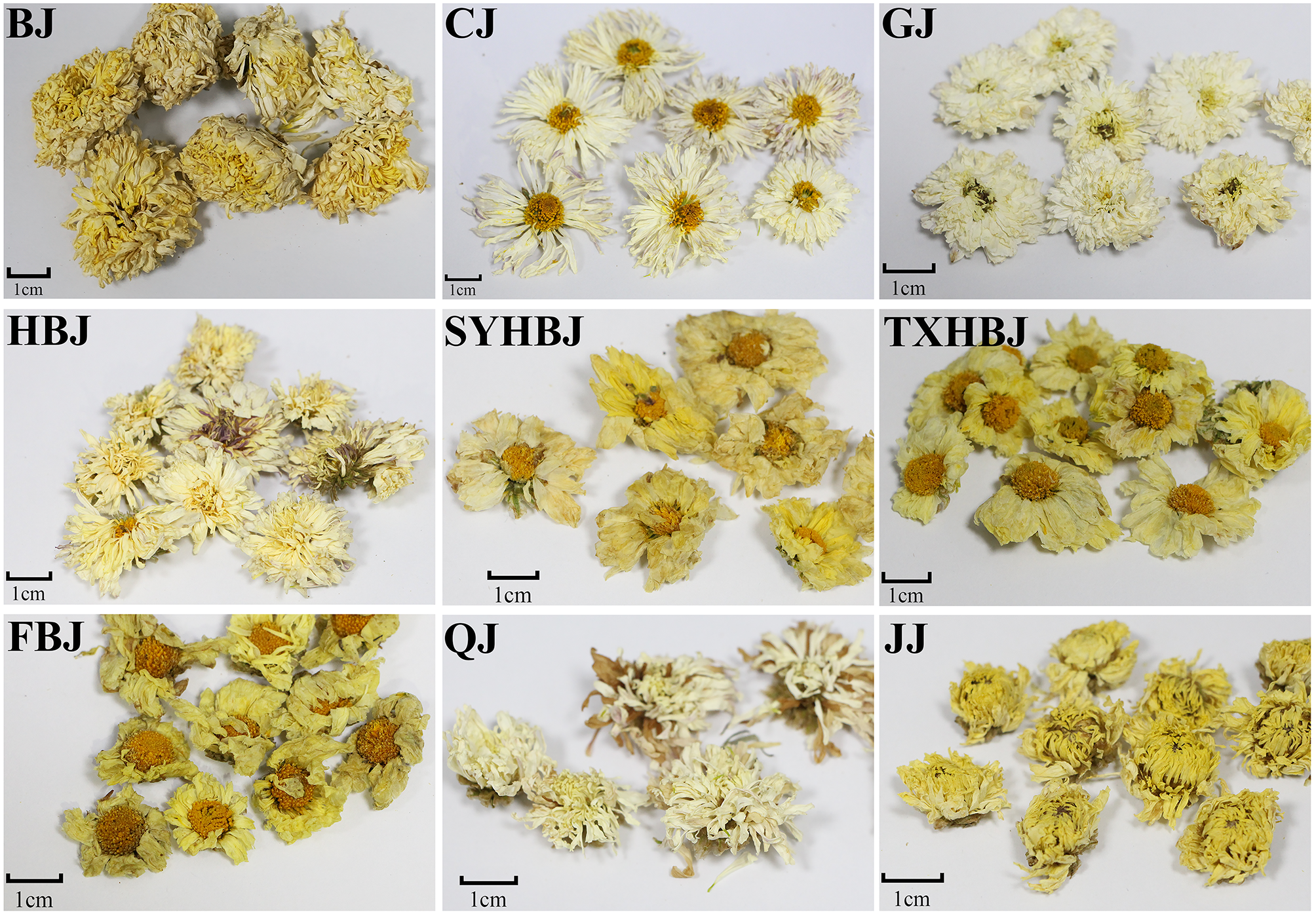


**Figure S2.** Digital photographs depicting Chrysanthemi Flos from nine different geographical origins.

**
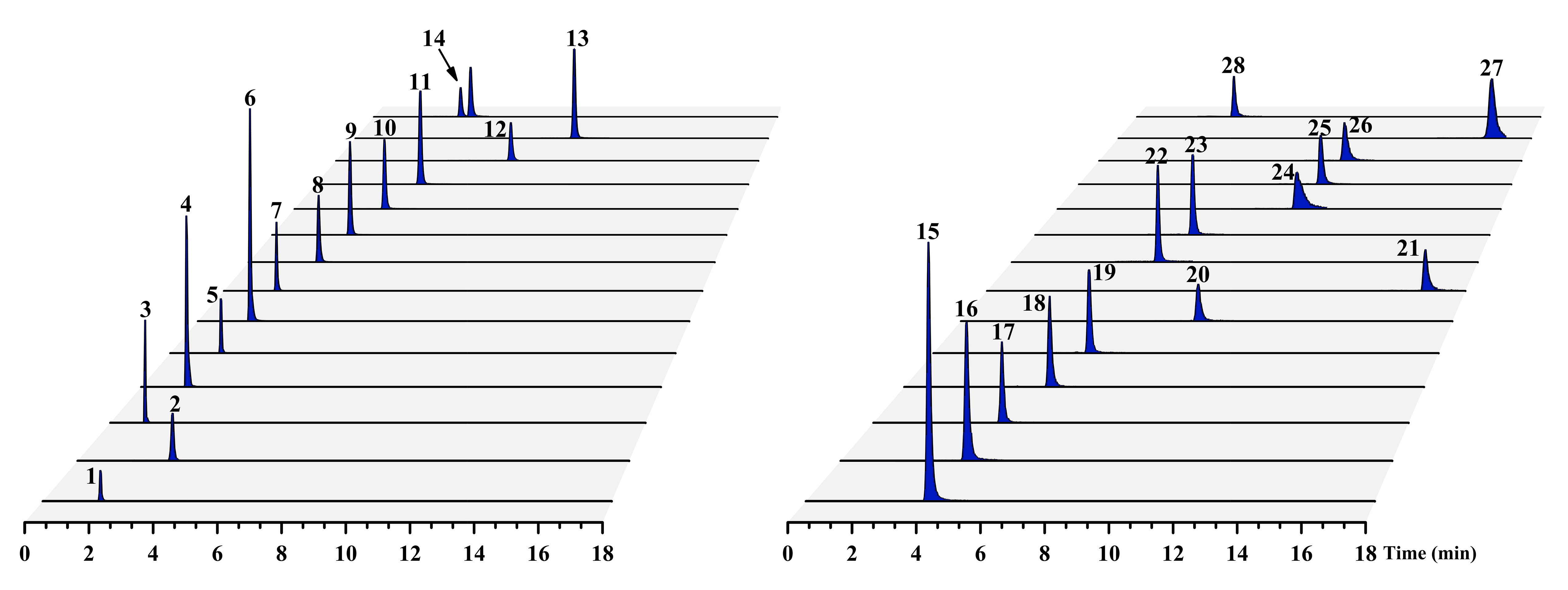
**

**Fi****gure S3.** Representative UPLC–MS/MS chromatograms of the 28 target compounds in mixed standards. The numbering of the 28 target compounds in the chromatograms is the same as in Figure S1.


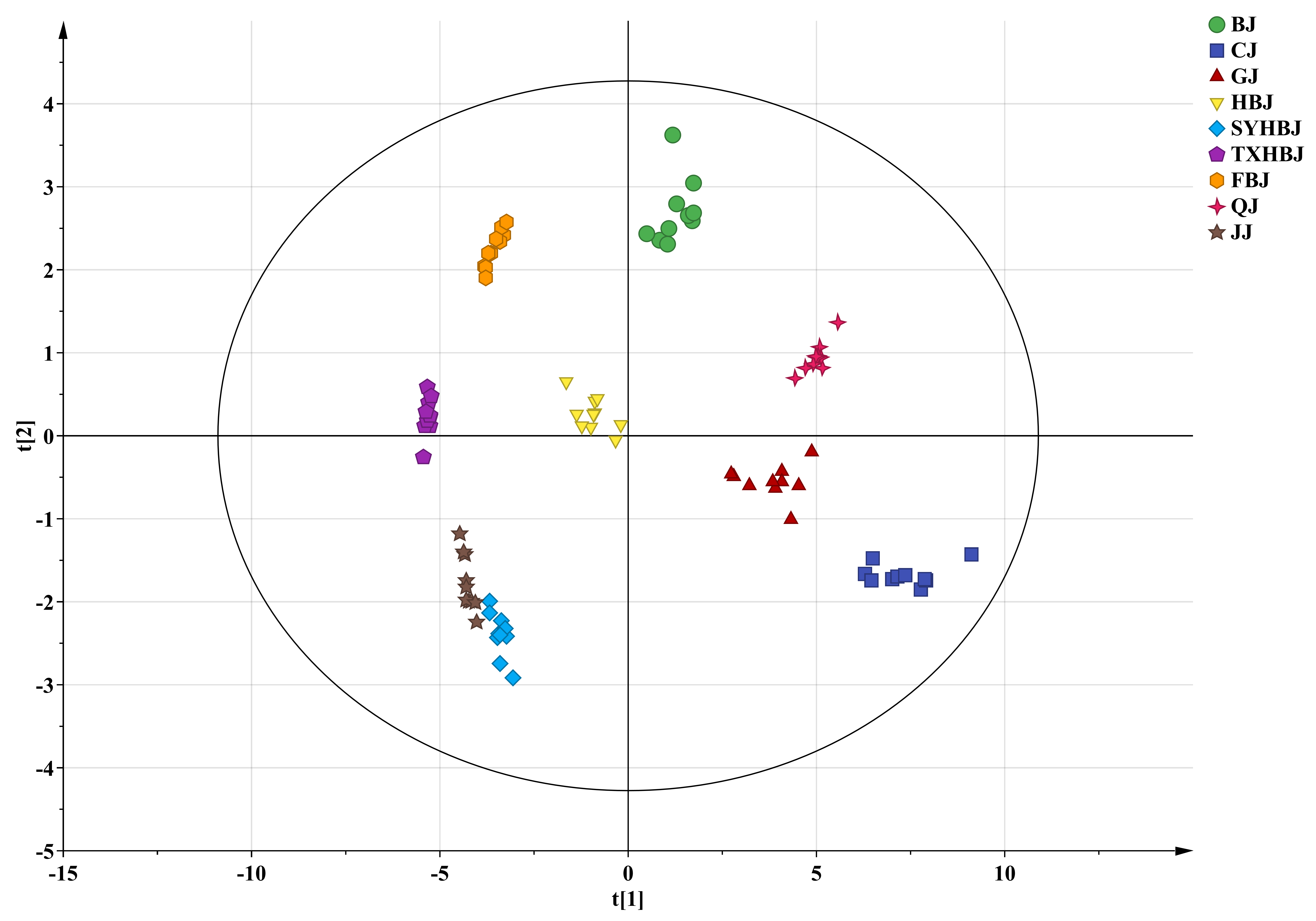


**Figure S4.** PCA score plot of Chrysanthemi Flos marked by nine different geographical origins.


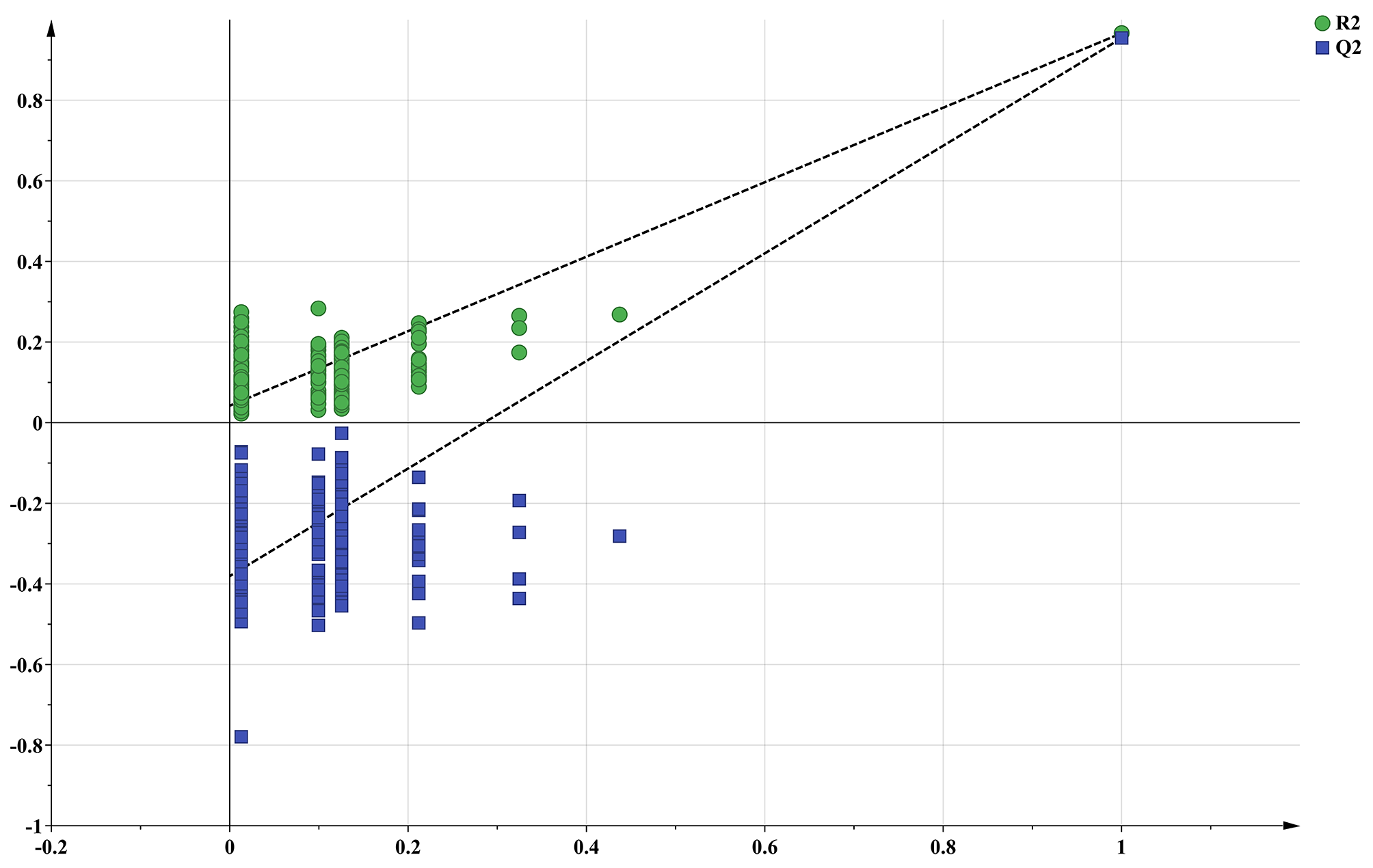


**Figure S5.** A presentation of 200 times permutation test for PLS-DA analysis.


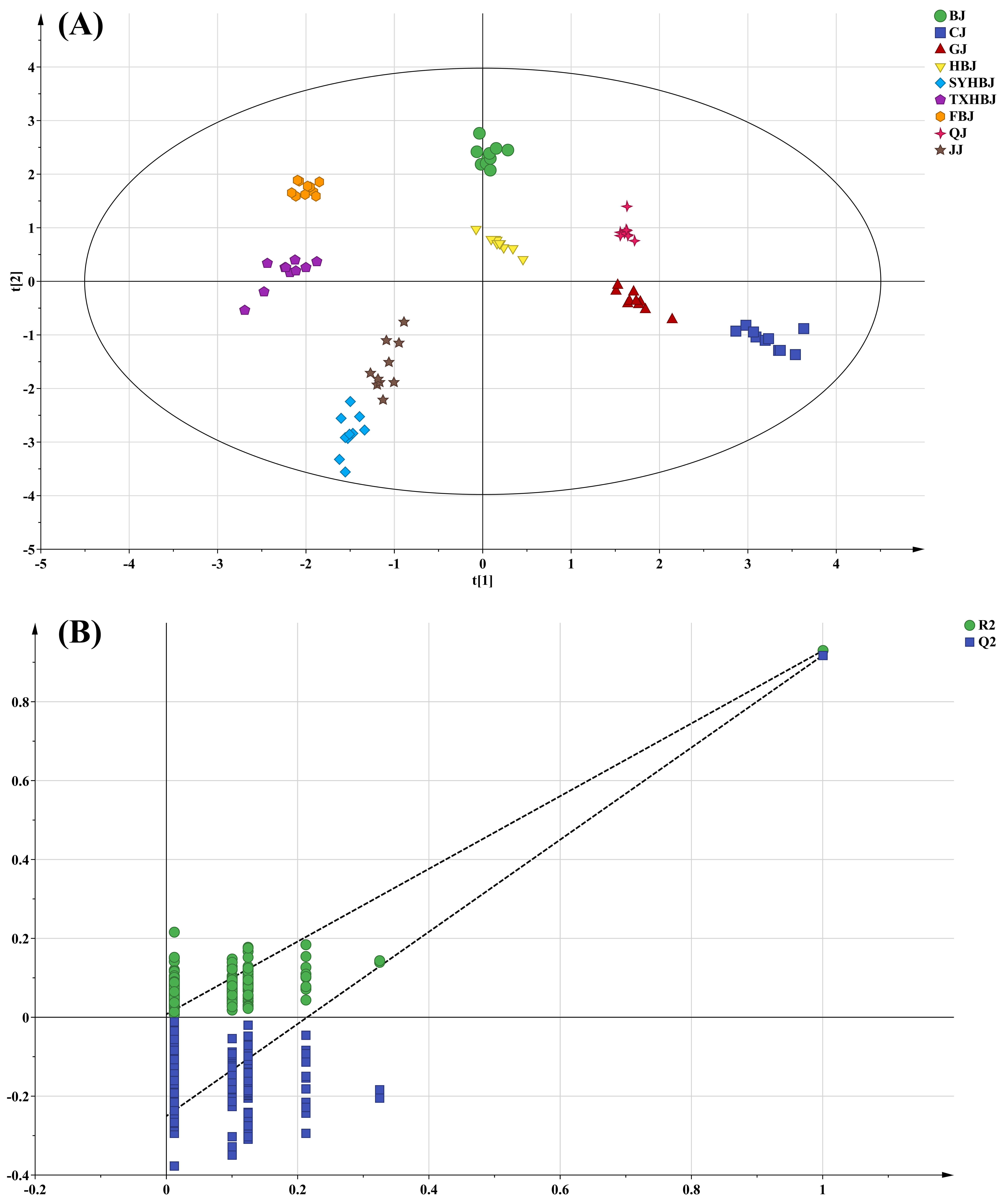


**Figure S6.** Multivariate statistical analysis of Chrysanthemi Flos from nine different geographical origins based on the screened eight quality markers: (A) PLS-DA score plot; (B) 200 times permutation test for PLS-DA model.

**Table S1.** Summary of the Chrysanthemi Flos samples harvested at different geographical origins.

| Sample number | Cultivars / Commercial name | Cultivation regions | Collection date | Longitude (E) | Latitude (N) | Elevation (m) |
| --- | --- | --- | --- | --- | --- | --- |
| S1-10 | Boju (BJ) | Bozhou, Anhui | November 3 | 115° | 33° | 43 |
| S11-20 | Chuju (CJ) | Chuzhou, Anhui | November 5 | 118° | 32° | 42 |
| S21-30 | Gongju (GJ) | Huangshan, Anhui | November 6 | 118° | 29° | 154 |
| S31-40 | Huaibaiju (HBJ) | Zhengzhou, Henan | October 25 | 113° | 34° | 92 |
| S41-50 | Sheyanghangbaiju (SYHBJ) | Sheyang, Jiangsu | November 9 | 120° | 33° | 2 |
| S51-60 | Tongxianghangbaiju (TXHBJ) | Tongxiang, Zhejiang | November 11 | 120° | 30° | 9 |
| S61-70 | Fubaiju (FBJ) | Macheng, Hubei | October 28 | 115° | 31° | 102 |
| S71-80 | Qiju (QJ) | Anguo, Hebei | October 20 | 115° | 38° | 31 |
| S81-90 | Jiaju (JJ) | Jiaxiang, Shandong | October 23 | 116° | 35° | 38 |

**Table S2.** Regression equation, correlation coefficients (r^2^), linearity ranges, LOD, LOQ, *F*-test, partial *F*-test and CVM test of 28 target compounds investigated by UPLC–MS/MS.

| **Analytes *^a^*** | **Calibration curves *^b^*** | **r^2^** | **Linear range (ng/mL)** | **LOD (ng/mL)** | **LOQ (ng/mL)** | ***F*-test** | **Partial *F*-test** | **CVM test** |
| --- | --- | --- | --- | --- | --- | --- | --- | --- |
| **1** | *y* = 2.51 *x* -623.55 | 0.9977 | 278.65–35666.67 | 10.96 | 36.53 | 1.42×10^-3^ | 1.07×10^-1^ | 0.978 |
| **2** | *y* = 306.93 *x* + 1181.72 | 0.9972 | 0.32–5200.00 | 0.08 | 0.27 | 3.15×10^-2^ | 5.76×10^-2^ | 0.943 |
| **3** | *y* = 57.59 *x* + 661.85 | 0.9996 | 1.14–75000.00 | 0.16 | 0.53 | 1.28×10^-7^ | 7.30×10^-2^ | 0.905 |
| **4** | *y* = 3851.76 *x* + 28441.47 | 0.9979 | 0.19–6145.83 | 0.06 | 0.18 | 5.10×10^-5^ | 6.14×10^-2^ | 0.714 |
| **5** | *y* = 24.48 *x* + 881.71 | 0.9990 | 14.77–121000.00 | 1.81 | 5.81 | 2.82×10^-8^ | 2.47×10^-1^ | 0.839 |
| **6** | *y* = 1717.48 *x* + 24753.39 | 0.9983 | 0.12–62666.67 | 0.03 | 0.10 | 1.17×10^-4^ | 8.05×10^-2^ | 0.468 |
| **7** | *y* = 82.89 *x* + 617.71 | 0.9997 | 1.15–4708.33 | 0.13 | 0.43 | 3.01×10^-2^ | 1.95×10^-1^ | 0.991 |
| **8** | *y* = 474.92 *x* + 1770.84 | 0.9992 | 0.14–9166.67 | 0.03 | 0.10 | 9.08×10^-8^ | 6.27×10^-1^ | 0.954 |
| **9** | *y* = 411.96 *x* + 4657.79 | 0.9993 | 1.28–10500.00 | 0.19 | 0.66 | 5.77×10^-5^ | 4.19×10^-1^ | 0.999 |
| **10** | *y* = 212.63 *x* + 784.96 | 0.9991 | 1.41–46333.33 | 0.20 | 0.69 | 8.02×10^-3^ | 8.05×10^-2^ | 0.943 |
| **11** | *y* = 291.39 *x* + 4593.48 | 0.9988 | 0.55–72166.67 | 0.09 | 0.29 | 5.69×10^-7^ | 9.12×10^-2^ | 0.967 |
| **12** | *y* = 281.12 *x* -5512.67 | 0.9985 | 32.88–33666.67 | 9.65 | 31.05 | 4.05×10^-6^ | 3.01×10^-1^ | 0.973 |
| **13** | *y* = 307.38 *x* -7324.83 | 0.9994 | 67.55–138333.33 | 5.19 | 16.89 | 3.26×10^-7^ | 5.47×10^-2^ | 0.957 |
| **14** | *y* = 60.36 *x* + 752.55 | 0.9985 | 1.90–62333.33 | 0.25 | 0.76 | 7.05×10^-4^ | 6.28×10^-2^ | 0.984 |
| **15** | *y* = 405.87 *x* -2838.89 | 0.9981 | 12.82–105000.00 | 2.75 | 9.47 | 2.07×10^-6^ | 4.04×10^-1^ | 0.991 |
| **16** | *y* = 121.90 *x* + 3505.93 | 0.9997 | 7.08–55940.30 | 1.56 | 5.56 | 9.04×10^-3^ | 8.59×10^-2^ | 0.792 |
| **17** | *y* = 25.36 *x* -4749.64 | 0.9994 | 614.58–157333.33 | 20.87 | 68.83 | 4.10×10^-2^ | 1.66×10^-1^ | 0.983 |
| **18** | *y* = 34.25 *x* -266.79 | 0.9996 | 15.38–63000.00 | 1.62 | 5.82 | 6.57×10^-3^ | 6.97×10^-2^ | 0.966 |
| **19** | *y* = 34.99 *x* -474.70 | 0.9991 | 24.66–101000.00 | 5.86 | 19.65 | 1.09×10^-7^ | 5.49×10^-2^ | 0.816 |
| **20** | *y* = 12.13 *x* -425.76 | 0.9979 | 118.16–121000.00 | 5.19 | 17.52 | 6.75×10^-4^ | 4.72×10^-1^ | 0.923 |
| **21** | *y* = 4.05 *x* + 6594.68 | 0.9997 | 0.77–50333.33 | 0.08 | 0.30 | 3.01×10^-8^ | 5.60×10^-2^ | 0.859 |
| **22** | *y* = 88.02 *x* -627.33 | 0.9994 | 19.53–800000.00 | 3.68 | 12.86 | 3.40×10^-5^ | 2.51×10^-1^ | 0.820 |
| **23** | *y* = 30.33 *x* + 735.19 | 0.9986 | 3.87–63333.33 | 0.57 | 1.94 | 7.52×10^-6^ | 9.71×10^-2^ | 0.983 |
| **24** | *y* = 3.92 *x* + 1602.29 | 0.9999 | 333.98–513000.00 | 15.43 | 51.77 | 2.20×10^-3^ | 7.95×10^-2^ | 0.679 |
| **25** | *y* = 4.70 *x* + 204.30 | 0.9975 | 69.66–428000.00 | 6.56 | 22.15 | 8.23×10^-8^ | 3.18×10^-1^ | 0.557 |
| **26** | *y* = 1.22 *x* + 2507.69 | 0.9993 | 52.49–860000.00 | 4.68 | 14.97 | 1.30×10^-5^ | 5.04×10^-1^ | 0.989 |
| **27** | *y* = 14.83 *x* + 27624.34 | 0.9988 | 120.77–494666.67 | 5.44 | 17.51 | 7.10×10^-8^ | 4.53×10^-1^ | 0.679 |
| **28** | *y* = 12.28 *x* -4598.23 | 0.9998 | 786.46–100666.67 | 22.62 | 86.65 | 2.34×10^-2^ | 3.77×10^-1^ | 0.953 |

*^a^* The 28 analytes are the same as in Figure S1.

*^b^ y* is the value of peak area, and *x* is the value of the reference compound’s concentration (ng/mL).

**Table S3.** Precision, repeatability, stability, recovery and matrix effect of 28 target compounds investigated by UPLC–MS/MS.

| Analytes *^a^* | Precision (RSD, %) | | Repeatability  (RSD, %, *n* = 6) | Stability  (RSD, %, *n* = 6) | Recovery (%, *n*=3) | | Matrix  effect *^b^* |
| --- | --- | --- | --- | --- | --- | --- | --- |
|  | Intra-day (*n*=6) | Inter-day (*n*=6) |  |  | Mean | RSD (%) |  |
| **1** | 1.96 | 2.83 | 1.52 | 3.50 | 96.41 | 2.98 | 0.95 |
| **2** | 1.66 | 4.14 | 3.20 | 1.79 | 94.40 | 2.80 | 0.97 |
| **3** | 3.04 | 5.93 | 1.59 | 2.28 | 102.50 | 4.30 | 1.05 |
| **4** | 2.15 | 4.06 | 2.50 | 5.11 | 100.50 | 1.64 | 0.94 |
| **5** | 0.81 | 1.23 | 4.46 | 3.36 | 97.06 | 2.15 | 0.92 |
| **6** | 2.41 | 2.75 | 3.15 | 1.58 | 103.10 | 5.10 | 0.95 |
| **7** | 3.62 | 4.16 | 1.40 | 4.06 | 97.14 | 3.83 | 1.05 |
| **8** | 3.43 | 4.27 | 2.17 | 1.58 | 97.20 | 4.45 | 0.98 |
| **9** | 1.10 | 4.10 | 5.21 | 3.53 | 103.30 | 2.70 | 0.95 |
| **10** | 3.30 | 5.10 | 5.30 | 2.61 | 96.74 | 4.12 | 1.03 |
| **11** | 1.97 | 2.36 | 1.62 | 4.25 | 94.30 | 2.32 | 0.98 |
| **12** | 1.48 | 5.54 | 3.38 | 3.06 | 97.21 | 3.80 | 0.96 |
| **13** | 3.21 | 3.49 | 6.84 | 3.34 | 102.11 | 5.60 | 0.98 |
| **14** | 2.39 | 3.02 | 2.61 | 6.69 | 96.60 | 3.65 | 1.02 |
| **15** | 1.48 | 1.75 | 3.66 | 1.46 | 104.75 | 3.40 | 1.03 |
| **16** | 2.69 | 4.58 | 3.14 | 5.22 | 94.79 | 1.97 | 0.91 |
| **17** | 1.15 | 2.66 | 1.64 | 2.95 | 103.63 | 4.87 | 0.93 |
| **18** | 2.82 | 5.49 | 5.17 | 4.95 | 95.40 | 5.27 | 1.02 |
| **19** | 3.53 | 6.24 | 3.95 | 1.59 | 96.70 | 2.75 | 0.95 |
| **20** | 1.83 | 2.67 | 2.75 | 6.25 | 103.60 | 2.05 | 1.06 |
| **21** | 0.98 | 1.21 | 1.90 | 3.98 | 95.05 | 3.17 | 0.92 |
| **22** | 1.60 | 2.70 | 3.94 | 4.72 | 94.34 | 1.76 | 0.94 |
| **23** | 2.50 | 4.80 | 4.87 | 1.70 | 104.60 | 4.23 | 0.95 |
| **24** | 1.80 | 3.90 | 1.83 | 5.02 | 95.80 | 1.60 | 1.06 |
| **25** | 1.90 | 3.70 | 6.14 | 3.87 | 95.85 | 4.15 | 0.93 |
| **26** | 1.28 | 3.02 | 4.02 | 5.48 | 100.16 | 3.03 | 0.92 |
| **27** | 1.38 | 1.94 | 1.24 | 2.47 | 103.84 | 3.58 | 0.99 |
| **28** | 1.25 | 2.77 | 5.15 | 3.02 | 95.70 | 4.15 | 1.04 |

*^a^* The 28 analytes are the same as in Figure S1.

*^b^* Matrix effects are calculated by slope matrix/slope solvent.
